# Mechanisms of Resistance to Epidermal Growth Factor Receptor Inhibitors and Novel Therapeutic Strategies to Overcome Resistance in NSCLC Patients

**DOI:** 10.1155/2012/817297

**Published:** 2012-08-29

**Authors:** Luping Lin, Trever G. Bivona

**Affiliations:** Division of Hematology/Oncology, Department of Medicine, USCF Helen Diller Family Comprehensive Cancer Center, University of California, San Francisco, San Francisco, CA 94158, USA

## Abstract

The epidermal growth factor receptor (EGFR) is a well-characterized oncogene that is frequently activated by somatic kinase domain mutations in non-small cell lung cancer (NSCLC). EGFR TKIs are effective therapies for NSCLC patients whose tumors harbor an EGFR activating mutation. However, EGFR TKI treatment is not curative in patients because of both primary and secondary treatment resistance. Studies over the last decade have identified mechanisms that drive primary and secondary resistance to EGFR TKI treatment. The elucidation of mechanisms of resistance to EGFR TKI treatment provides a basis for the development of therapeutic strategies to overcome resistance and enhance outcomes in NSCLC patients. In this paper, we summarize the mechanisms of resistance to EGFR TKIs that have been identified to date and discusses potential therapeutic strategies to overcome EGFR TKI resistance in NSCLC patients.

## 1. Introduction

Lung cancer is the leading cause of cancer mortality in the United States and worldwide, accounting for 28% of cancer-related deaths in males and 26% of cancer-related deaths in females [1, 2]. Most lung cancer patients present with advanced stage disease, for which conventional chemotherapies patients are only modestly effective. Thus, the 5-year-survival rate of lung cancer patients with metastatic disease is less than 15% [3]. In the last decade, the discovery of mutated oncogenes that encode activated signaling molecules that drive cellular proliferation and promote tumor growth has led to the development of more effective and less toxic targeted drugs for lung cancer patients. Systemic therapies that act against specific activated oncogenes in lung cancers have the potential for improving outcomes for lung cancer patients in an unprecedented manner. Yet, a significant challenge that must be overcome in order to realize the full potential of targeted cancer therapy in lung cancer patients is resistance to treatment with an oncogene inhibitor as monotherapy.

The epidermal growth factor receptor (EGFR) is a well-characterized mutated oncogene in non-small cell lung cancer (NSCLC) that is found in ~10–20% of cases in western countries and is associated predominantly with adenocarcinoma histology. EGFR-mutated tumors are dependent to EGFR signaling for their proliferation and survival [4–7]. In lung cancer patients, EGFR mutations are generally exclusive with KRAS and BRAF mutations, and tumors with either KRAS (15–25%) or BRAF (2-3%) mutations are relatively insensitive to EGFR TKIs [8, 9]. The most common activating mutations (~90%) are in-frame deletions in exon 19 of EGFR and a missense mutation at 858 in exon 21 of EGFR resulting in an arginine to leucine substitution (L858R) [10]. Therapeutic agents targeting the EGFR signaling pathway, including two EGFR kinase inhibitors gefitinib and erlotinib, are clinically effective in treating lung cancer patients harboring these EGFR activating mutations [11–14].

Despite the dramatic efficacy of EGFR TKIs in NSCLC patients with EGFR activating mutations, unfortunately, de novo resistance to TKIs is observed and virtually all patients who initially respond will ultimately develop acquired resistance. In this paper, we focus on the mechanisms of both de novo resistance (lack of an initial response to therapy) and acquired resistance (resistance that develops following an initial response to therapy) to EGFR TKIs. We also discuss potential strategies to overcome resistance in lung cancer patients. It is currently not known whether acquired resistance occurs through clonal selection of resistant tumor cells present in the initial tumor or is induced during therapy. Approaches such as lineage tracing or next generation deep sequencing at the single-cell level could be used to address this unresolved issue.

## 2. De Novo Resistance to EGFR TKIs

Non-small cell lung cancers harboring an EGFR activating mutation can show primary resistance to EGFR TKI therapy. Among patients with an EGFR activating mutation, approximately 70% of them will experience significant tumor regressions when treated with an EGFR TKI [15–17]. Thus, approximately 30% of patients with an EGFR activating mutation experience de novo resistance to EGFR TKIs. Two general mechanisms of de novo resistance to EGFR TKI treatment in EGFR mutant NSCLC patients have been described to date: (1) secondary alterations in EGFR that prevent inhibition of EGFR by an EGFR TKI (drug resistant EGFR mutation), and (2) additional genetic alternations that can co-occur with an EGFR activating mutation in EGFR mutant NSCLC cells.

### 2.1. Drug Resistant EGFR Mutation

NSCLCs harboring a small insertion or duplication in exon 20 observed in ~5% of NSCLCs are less sensitive to EGFR TKIs compared to the exon 19 deletion mutants and L858R mutants in vitro [18], as well as in patients [19]. Similarly, patients harboring an EGFR T790M mutation in exon 20 are also resistant to EGFR TKI treatment [20–22]. Interestingly, the EGFR T790M mutation can also be found at low frequency (approximately 0.54% of never smokers with lung cancer) in the germ line of patients. The presence of a germline EGFR T790M mutation may be associated with increased risk of developing lung cancer [23–25]. In pretreated patients harboring a T790M mutation, low expression of BRCA1 mRNA is correlated with a prolonged progression-free survival to erlotinib treatment. The data suggest that low BRCA1 level may neutralize the negative effects of the EGFR T790M mutation on erlotinib sensitivity and that high BRCA1 expression may lead to de novo EGFR TKI resistance potentially through increased DNA damage repair capacity [26]. In addition to EGFR T790M, primary EGFR TKI resistance may also be due to other secondary mutations in EGFR (e.g., D761Y) that occur in cis with an activating EGFR kinase domain mutation (e.g., L858R) [27].

### 2.2. Other Genetic Alternations with EGFR Mutations

The presence of other genetic alterations together with EGFR activating mutations in lung cancer cells could lead to EGFR TKI resistance by promoting cell survival in the face of EGFR inhibition. Additional genetic alterations that promote EGFR TKI resistance that have been reported to date are summarized in the following discussion.

#### 2.2.1. Activation of Phosphoinositide-3-Kinase(PI3K)/AKT Signaling

Loss of PTEN expression in EGFR mutant cells has been associated with decreased sensitivity to EGFR TKIs by activation of PI3K-AKT signaling, the impairment of the ligand-induced ubiquitination, and degradation of activated EGFR [28, 29]. Moreover, PIK3CA, the p110alpha catalytic subunit of PI3K, was found to be mutated in approximately 1.3% of Japanese lung cancer patient with EGFR mutations versus 2.1% in patients without EGFR mutations [30]. In addition, the constitutively activated PI3K (E545K) has been shown to confer resistance of EGFR TKI in vitro [31].

#### 2.2.2. Involvement of IGF1R Signaling

**Figure 1:**
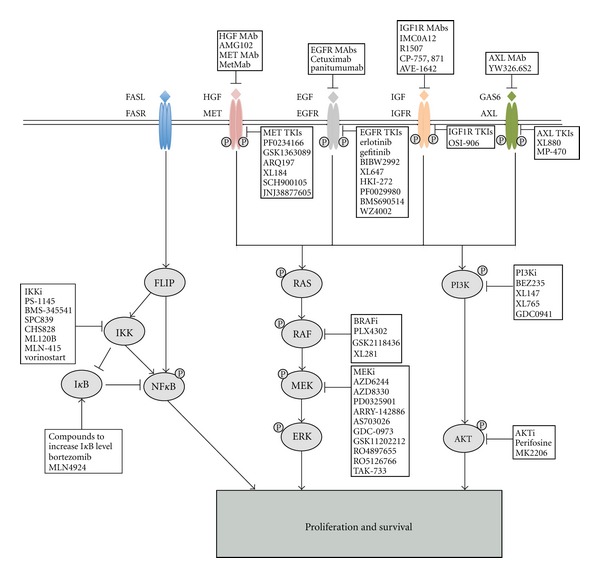
Mechanisms of resistance to EGFR TKIs and multiple strategies to overcome resistance in EGFR mutant lung cancer. EGFR signals through the RAS/RAF/MEK/ERK and PI3K/AKT pathways to promote cellular proliferation and survival. Crosstalk of other receptor tyrosine kinase confers resistance to EGFR TKIs by activation of both MAPK and AKT signaling. The FAS/NF*κ*B signaling arm downstream of FAS death receptor also contributes to resistance to EGFR TKIs. Available targeted agents that act against pathways that drive EGFR TKI resistance and that may overcome resistance to EGFR TKIs in appropriately selected lung cancer patients are shown.

The IGF1R signaling pathway has been implicated in EGFR TKI resistance through crosstalk with EGFR signaling in cell lines harboring EGFR activating mutations. For example, cotreatment of erlotinib and an IGF1R inhibitor induced both apoptosis and cell cycle arrest, while single agent of either inhibitor alone only induce cell cycle arrest in some EGFR mutant NSCLC cells [32]. Moreover, Sharma et al. discovered that EGFR mutant lung cancer cell lines persisting after EGFR TKI treatment were enriched for a drug-tolerant subpopulation that may have existed prior to treatment. This drug-tolerant subpopulation of cells showed a distinct chromatin state that is regulated by IGF1R signaling and histone demethylase [33] (Figure 1).

#### 2.2.3. Activation of NF*κ*B Signaling

NF*κ*B signaling has been broadly associated with inflammation and cancer [34]. Recently, Bivona et al. identified activation of NF*κ*B signaling as a new mechanism of de novo resistance to erlotinib treatment. This group used a high throughput unbiased pooled shRNA screening approach in H1650 cells that harbor an exon 19 deletion in EGFR and are insensitive to EGFR TKI treatment to define genetic modifiers that contribute to de novo EGFR TKI resistance. Of the 36 shRNAs recovered from the pooled screen, 18 targeted genes that are involved in NF*κ*B signaling directly or indirectly. Interestingly, one of the top hits in the pooled screen was CD95/FAS, the ligand of the FAS death receptor. Prior work had shown that CD95/FAS can, in some contexts, function upstream of NF*κ*B to promote cell survival and tumor growth. The authors found that genetic or pharmacologic inhibition of NF*κ*B signaling increased sensitivity to erlotinib but not to chemotherapy in several models of EGFR mutant lung cancer. Intriguingly, low expression of the NF*κ*B inhibitor I*κ*B was predictive of a poor clinical outcome in patients treated with erlotinib without a T790M mutation. Importantly, I*κ*B status was not predictive of outcomes in EGFR mutant lung cancer patients treated with surgery or chemotherapy, indicating NF*κ*B signaling is specific biomarker of EGFR TKI response in this patient population [35, 36] (Figure 1). These results suggest that hyperactivation of NF*κ*B signaling may cause de novo resistance to EGFR TKI treatment in EGFR mutant lung cancer patients.

## 3. Acquired Resistance to EGFR TKIs

Among NSCLC patients with activating EGFR mutation, approximately 70% will experience significant tumor regressions when treated with an EGFR TKI [15–17]. However, the vast majority of patients that initially respond to EGFR TKI treatment develop acquired EGFR TKI resistance after a median of 10–14 months on EGFR-TKI treatment [11, 14, 37].

### 3.1. Second-Site Mutations

Approximately 50% of patients with EGFR mutant lung cancers who develop acquired resistance to EGFR TKIs have a second-site mutation T790M in the threonine gatekeeper residue that coexists with a primary EGFR activating mutation [38, 39]. The T790M mutation occurs at a conserved threonine residue located near the kinase active site that is found in many kinases and is often referred to as the “gatekeeper mutation”. Gatekeeper mutations have been found in many kinase-driven tumors that develop resistance to kinase inhibitor treatment (e.g., CML patients treated with imatinib that develop the T315I resistance mutation in BCR-ABL). Two potential mechanisms could explain how the EGFR T790M mutation confers EGFR TKI resistance. One possibility is that the T790M mutation could lead to altered drug binding in the ATP pocket of EGFR in a manner analogous to the effects of the T315I mutation in the ABL kinase in the context of imatinib resistance [40]. The other possibility is that the presence of the EGFR T790M mutation could increase the affinity of the EGFR-L858R for ATP and thus reduce the potency of an ATP-competitive kinase inhibitor [41]. Alternative pharmacological strategies targeting EGFR T790M may be therapeutically efficacious to treat patients with acquired resistance to EGFR TKIs and an EGFR T790M secondary mutation.

The EGFR T790M mutant exhibits synergistic kinase activity and transformation potential when coexisting with an EGFR activating mutation in preclinical models [42, 43]. Interestingly, the subclonal populations of EGFR mutant tumor cells with and without the EGFR T790M allele may coexist in an EGFR mutant lung cancer with acquired EGFR TKI resistance. This clonal heterogeneity may explain both the “flare” phenomenon (rapid tumor regrowth upon withdrawal of an EGFR TKI) observed in EGFR mutant lung cancer patients upon discontinuation of an EGFR TKI and also the finding that patients may respond to subsequent EGFR TKI treatment after initial discontinuation of therapy. [44–47]. The detailed biological mechanisms underlying these clinical phenomena are unknown.

In addition to the EGFR T790M mutation, there are three other second-site mutations in EGFR that have been associated with acquired EGFR TKI resistance: T854A in exon21 [48], L747S [49], and D761Y [27], both in the exon19. Similar to the EGFR T790M mutation, alteration of the drug contact residue T854 to a smaller hydrophobic alanine may increase the size of the selectivity pocket and negatively impact erlotinib binding. L747S is thought to shift the equilibrium towards the active conformation of the receptor, while D761Y may affect the catalytic cleft of the receptor. Both T854 and D761 were identified in laboratory models of erlotinib resistance in addition to clinical samples [50].

### 3.2. MET Amplification

Amplification of MET, a receptor tyrosine kinase, was detected in up to 20% of lung cancer specimens that developed acquired resistance to gefitinib or erlotinib. Although MET amplification can coexist with the EGFR T790M mutation, approximately 60% of MET amplification is independent of T790M mutation [51, 52]. MET amplification was originally identified in a laboratory-model of gefitinib resistance using HCC827 human EGFR mutant NSCLC cells. In this model, cells with EGFR TKI resistance relied on MET signaling to activate AKT through ERBB3-mediated activation of PI3K in the presence of EGFR TKIs [52]. In addition, the MET ligand hepatocyte growth factor (HGF) induced gefitinib resistance through activation of MET-PI3K signaling [53]. MET amplification was also observed at a low frequency in EGFR mutant lung cancers in patients prior to treatment and was associated with the development of acquired resistance to EGFR TKIs [54]. Together the data suggest that EGFR TKI treatment may select for preexisting cells with MET amplification during the acquisition of EGFR TKI resistance (Figure 1).

### 3.3. Other Potential Mechanisms

EGFR T790M and MET amplification account for ~60% of acquired resistance to EGFR TKIs. Other mechanisms of resistance that are operative in the remaining ~40% of tumors with acquired resistance to EGFR TKIs are under active investigation.

#### 3.3.1. PI3KCA Mutation

Recently, mutations in PIK3CA were identified in ~5% of EGFR mutant lung cancers that developed acquired EGFR TKI resistance. These clinical data were consistent with earlier data demonstrating that introduction of an activated PIK3CA mutant into the EGFR mutant cell line HCC827 confers resistance to gefitinib [31, 55]. These findings suggest that lung cancer patients with both EGFR and PIK3CA mutations could be considered for combination therapy with an EGFR TKI and a PI3K inhibitor.

#### 3.3.2. EMT and Histological Transformation

The epithelial to mesenchymal transition (EMT) has been considered as a general biological switch rendering NSCLC sensitive or insensitive to EGFR inhibition [56, 57]. Increased expression of E-cadherin, an epithelial marker, has been associated with clinical activity of EGFR inhibitors in NSCLC patients [58, 59]. EMT has been also associated with acquired resistance of EGFR TKIs in laboratory models [60, 61]. Recently, several studies showed that EMT was observed in EGFR mutant lung cancers with acquired resistance to EGFR TKIs (2 of 37) [55]. It is not known if mesenchymal-like cells in the acquired resistant tumors are exist prior to therapy or are induced upon drug treatment.

Sequist et al. unexpectedly found a histological transformation from NSCLC into small cell lung cancer (SCLC) in 14% of EGFR mutant lung cancer patients (5 of 37) with acquired EGFR TKI resistance. Importantly, transformation to SCLC was associated with a response to treatment with standard SCLC chemotherapy. Three independent studies have identified three cases of SCLC harboring an EGFR mutation [62–64]. Further investigation is necessary to clarify the role and genesis of these histological transformations in EGFR inhibitor resistance in lung cancer patients.

#### 3.3.3. Activation of AXL

Recently, three groups independently discovered that activation of the AXL receptor tyrosine kinase confers acquired resistance to erlotinib in both cell culture and tumor xenograft models of EGFR mutant lung cancer. Activation of AXL occurred through overexpression as well as through upregulation of its ligand GAS6 in the setting of EGFR TKI resistance in EGFR-mutant NSCLCs. Genetically or pharmacologically inhibiting AXL restored erlotinib sensitivity both in vitro and in vivo. Moreover, forced expression of AXL in EGFR mutant lung cancer cell lines that are sensitive to erlotinib induced erlotinib resistance through the kinase activity of AXL. Interestingly, upregulation of AXL was associated with the development of an epithelial to mesenchymal transition (EMT) in EGFR mutant tumors with acquired erlotinib resistance. By comparing expression of AXL in pre- and posterlotinib treatment patients, approximately 20% of the EGFR TKI resistance cases showed increased AXL expression. This observation provides strong rationale for the development and testing of AXL kinase inhibitors for clinical use in EGFR-mutant NSCLC patients to either prevent or overcome acquired EGFR TKI resistance [65] (Figure 1).

#### 3.3.4. Other Mechanisms: IGF1R and PTEN Pathways

Increased IGF1R signaling through the loss of inhibitory IGF-binding proteins has also been associated with acquired EGFR TKI resistance in a laboratory model using a lung squamous cell line expressing high-level of wild-type EGFR [66]. Loss or reduction of the tumor suppressor PTEN has been associated with acquired EGFR TKI resistance in laboratory models [67, 68]. However, hyperactive IGF1R and PTEN loss have not been yet validated as mechanisms of acquired EGFR TKI resistance in clinical specimens.

**EGFR-Directed Antibody Therapy**

In addition to EGFR TKIs, the EGFR-directed antibody cetuximab is also an effective clinical therapy for patients with NSCLC, colorectal, and head and neck cancers. Some NSCLC cells that are sensitive to EGFR TKIs are sensitive to cetuximab as well. EGFR mutant HCC827 cells with acquired resistance to cetuximab were generated and shown to harbor amplification of ERBB2 as a mechanism of EGFR TKI resistance. Inhibition of ERBB2 or disruption of ERBB2/ERBB3 heterodimerization restored cetuximab sensitivity in vitro and in vivo in these models [69].

To date, the biological basis underlying acquired EGFR TKI resistance is unknown in ~30% of patients. Some mechanisms of resistance that have been identified using laboratory models have not been validated in patients with acquired resistance, indicating the limitation of these laboratory models of acquired EGFR TKI resistance. Integrated approaches to study acquired EGFR TKI resistance using genetically engineered mouse models (GEMMs) of EGFR mutant lung cancer combined with patient derived cell lines and the analysis of clinical specimens hold promise for deciphering the remaining unknown mechanisms of EGFR TKI resistance [70].

## 4. Overcoming Resistance to EGFR TKIs

### 4.1. De Novo Resistance

#### 4.1.1. Novel EGFR TKIs

For lung cancer patients harboring a secondary mutation in EGFR that abrogates EGFR TKI affinity or binding, such as exon 20 insertion, duplication and the T790M substitution, the development of novel EGFR TKIs are needed to more effectively target mutant EGFR. The second-generation EGFR TKI PF00299804 (Pfizer) has been shown to induce partial response in one patient with an EGFR exon 20 insertion [71]. Moreover, the second-generation irreversible EGFR inhibitors were shown in preclinical models to be more potent targeting T790M mutation than gefitinib or erlotinib [72]. Emerging EGFR TKIs that exhibit increased potency against an activated EGFR mutant oncoprotein may open a therapeutic window for patient with rare mutations of EGFR.

#### 4.1.2. Polytherapies

For patients harboring other genetic alterations along with an EGFR activating mutation, polytherapies could be pursued. For example, NF*κ*B signaling can decrease erlotinib sensitivity in NSCLC cells with EGFR activating mutation, leading to de novo resistance to EGFR TKI treatment [35]. Compounds such as MLN0415 and BMS345541 that target IKK and IKK-related kinases (e.g., IKK*β*) that activate NF*κ*B [73] may overcome de novo resistance to EGFR TKI treatment. Similarly, compounds such as Nedd8 activating enzyme (NAE) inhibitor MLN4924 or the proteasome inhibitor bortezomib that lead to increased levels of the NF*κ*B inhibitor I*κ*B [74, 75] may increase responses to EGFR TKI treatment.

EGFR mutant lung cancers with genetic alterations that activate the PI3K-AKT signaling pathway or IGF1R signaling may benefit from treatment with PI3K or AKT inhibitors or an IGF1R antibody, respectively, in combination with an EGFR TKI (Figure 1). Three independent studies have shown that induction of the proapoptotic protein Bim is essential for apoptosis triggered by EGFR TKI treatment. Moreover, a polymorphism in BIM that generates a dysfunctional form of the protein that leads to intrinsic EGFR TKI resistance in EGFR mutant NSCLC cell lines was recently described. Together, the data suggest that adding a BCL-2 inhibitor (ABT-737) to EGFR TKI therapy could enhance responses in patients, particularly those patients with a BIM polymorphism. [76–79].

### 4.2. Acquired Resistance

#### 4.2.1. Irreversible EGFR TKIs

Approximately 50% of patients with acquired EGFR TKI resistance harbor a secondary T790M mutation in EGFR. Second-generation irreversible EGFR inhibitors, which bind irreversibly in the ATP-binding pocket of EGFR through a covalent bond at C797, were shown to be more potent inhibitors of the second-site T790M mutation than erlotinib or gefitinib in pre-clinical models [72, 80] (Figure 1). These inhibitors are currently under clinical trials in patients with acquired resistance. One of these agents, BIBW2992 (afatinib), is able to target both EGFR and ERBB2 and overcome T790M-driven acquired resistance [81]. However, in clinical studies, BIBW2992 did not prolong survival compared to placebo in NSCLC patients who have developed acquired resistance to gefitinib or erlotinib [82]. Another agent in this class of next generation EGFR TKIs, PF-002999804, inhibits all ERBB family members and has been shown to be effective against tumors harboring T790M [83, 84]. Unfortunately, resistance of EGFR T790M positive tumors to PF00299804 was developed rapidly through amplification of the EGFR T790M containing allele. This observation has hampered further clinical development of this agent. Chemical-genomic profiling studies indicate that the clinical utility of these irreversible EGFR TKIs is likely to be limited by the increased potency of these agents against both WT and mutant EGFR and, thus, the narrow therapeutic window of these irreversible inhibitors in the clinic [85].

#### 4.2.2. T790M Specific Inhibitors

Recently WZ4002, a new inhibitor specifically targeting T790M gatekeeper mutation was developed and found to induce greater growth inhibition in vitro and in vivo against T790M than against wild-type EGFR [86] (Figure 1). These findings indicate that the specificity of this class of inhibitors against the EGFR T790M oncoprotein may provide the ability to achieve clinical concentrations sufficient to effectively target tumor cells that express EGFR T790M and spare cells that express WT EGFR. Several agents in this promising class of EGFR TKIs are currently under clinical development.

#### 4.2.3. Polytherapies

In selected cases, polytherapies targeting compensatory pathways that lead to acquired EGFR TKI resistance may overcome resistance. For example, adding a MET inhibitor may be beneficial to EGFR mutant lung cancer patients whose tumors harbor MET amplification as a mechanism of EGFR TKI resistance. Antibodies targeting the MET ligand HGF (AMG102), MET itself (MetMAb), and small molecule inhibitors against MET are currently in clinical development (Figure 1). Moreover, due to the importance of AXL signaling in acquired resistance to EGFR TKI, the combination of small molecule kinase inhibitors targeting AXL (XL880 or MP-470) or an AXL neutralizing antibody with an EGFR TKI is also a potential approach to overcome resistance (Figure 1). Furthermore, several biological mechanisms of acquired resistance converge on activation of the PI3K/AKT and also MAPK signaling pathways. These observations provide rational to combine EGFR inhibitors with inhibitors of these pathways. For example, combination therapy with an irreversible EGFR inhibitor and an inhibitor of mTOR (rapamycin) lead to significant regression of tumors in genetic engineered lung tumor model driven by an EGFR T790M/L858R mutant oncoprotein [87]. Moreover, dual inhibition of EGFR with an irreversible EGFR inhibitor (afatinib) and the EGFR neutralizing antibody cetuximab has recently shown promising activity in EGFR mutant lung cancers with acquired EGFR TKI resistance that harbor an EGFR T790M mutation [88].

Recently, a clinical trial was conducted to investigate whether there would be any additional clinical benefit with the addition of systemic chemotherapy to an EGFR TKI in lung cancer patients. In the subgroup of 66 patients with an EGFR mutation who received either single-agent erlotinib or concurrent combination of chemotherapy and erlotinib, progression free survival was 15.7 and 17.2 months, respectively, in this trial. The data indicate that the addition of chemotherapy to erlotinib did not appear to improve treatment outcomes in patients with EGFR mutation [89].

#### 4.2.4. Alternative EGFR TKI Dosing and Continuation Therapy

A recent study incorporating mathematical modeling suggested that different dosing schedules of EGFR TKIs could significantly prolong the time to relapse without compromising efficacy [90]. Furthermore, continuation therapy that incorporates cycling EGFR TKI treatment may suppress the outgrowth of aggressive drug resistant clones that can be associated with the clinical phenomenon of tumor flare upon discontinuation of EGFR TKI therapy [44]. Based in part on these data, there is biological rationale for the continuation of EGFR TKI treatment upon tumor progression in patients. Thus, continuation and alternative EGFR TKIs dosing schedules and rational combination therapies are under active investigation to determine if different dosing regiments can significantly prolong the duration of response to EGFR TKI treatment in patients.

## 5. Summary

The ultimate goal of investigations that aim to understand the mechanisms of de novo and acquired EGFR TKI resistance is to allow us to design rational strategies to overcome resistance or to prevent resistance from developing altogether in patients. The characterization of the biological basis of EGFR TKI resistance will hopefully pave the way for novel therapeutic strategies to optimize responses to EGFR inhibition in EGFR mutant lung cancer patients by delaying or preventing the emergence of dominant drug resistant subclones that exist or are induced in an EGFR mutant lung cancer. Systematic and comprehensive interrogation of the genetic, epigenetic, and genomic alterations that drive the development of resistance to EGFR TKI treatment are underway and should yield rapid and substantial advances that lead to improved therapeutic strategies and survival outcomes for lung cancer patients.
